# Exploring unconventional targets in myofibroblast transdifferentiation outside classical TGF- β signaling in renal fibrosis

**DOI:** 10.3389/fphys.2024.1296504

**Published:** 2024-05-14

**Authors:** Rashida Lathan

**Affiliations:** School of Cardiovascular and Metabolic Health, College of Medical, Veterinary and Life Sciences, University of Glasgow, Glasgow, United Kingdom

**Keywords:** fibrosis, myofibroblasts, fibroblast transition, extracellular matrix, kidney, renal fibrosis, TGF-beta, transdifferentiation

## Abstract

We propose that the key initiators of renal fibrosis are myofibroblasts which originate from four predominant sources—fibroblasts, pericytes, endothelial cells and macrophages. Increased accumulation of renal interstitial myofibroblasts correlates with an increase in collagen, fibrillar proteins, and fibrosis severity. The canonical TGF- β pathway, signaling via Smad proteins, is the central molecular hub that initiates these cellular transformations. However, directly targeting these classical pathway molecules has proven challenging due their integral roles in metabolic process, and/or non-sustainable effects involving compensatory cross-talk with TGF-β. This review explores recently discovered alternative molecular targets that drive transdifferentiation into myofibroblasts. Discovering targets outside of the classical TGF-β/Smad pathway is crucial for advancing antifibrotic therapies, and strategically targeting the development of myofibroblasts offers a promising approach to control excessive extracellular matrix deposition and impede fibrosis progression.

## Introduction

Classically, the process of fibrosis in the kidney is broken down into five distinct stages.

**FIGURE 1:**
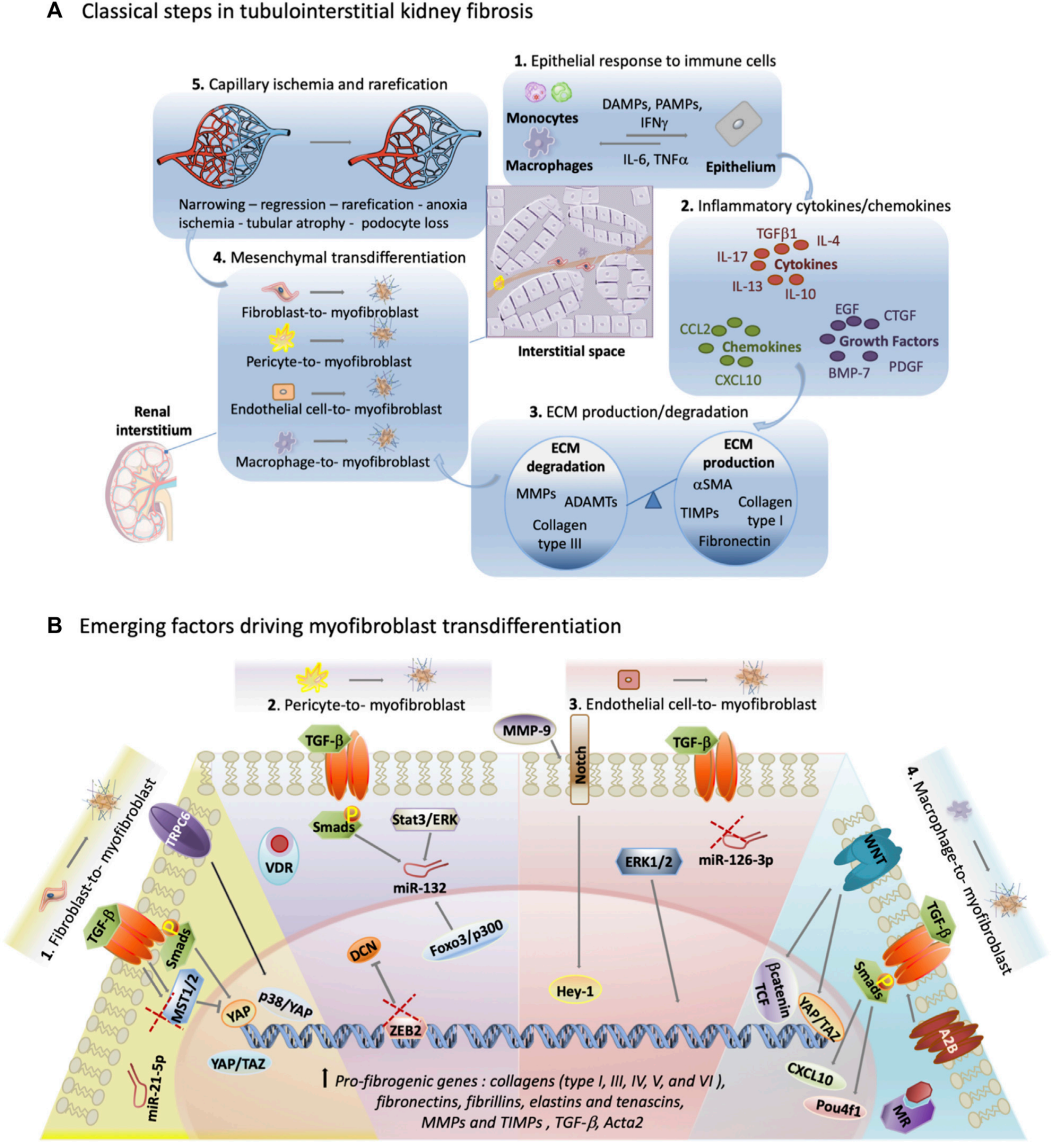
Exploring unconventional targets in myofibroblast transdifferentiation outside classical TGF- β signaling in renal fibrosis. **(A)** Illustration representing classical tubulointerstitial kidney fibrosis steps. DAMPs: Damage-associated molecular patterns, PAMPs: Pathogen-associated molecular pattern molecules, IFNγ: Interferon gamma. A portion of the figure utilizes adapted images from Servier Medical Art, licensed under Creative Commons Attribution 4.0 Unported License. **(B)**. Summary of emerging targets that cross-talk with TGF- β outside of the canonical TGF- β /Smad signaling cascade. Alternative molecular targets illustrated drive transdifferentiation into myofibroblasts from origin cells: (1) fibroblasts, (2) pericytes, (3) endothelial cells and (4) macrophages. Double arrow signifies prolonged and increased signaling. Red X indicates when removal of a potential target drives cellular transformation. TGF- β : Transforming growth factor-beta, TRPC6: transient receptor potential cation channel subfamily C member 6, Smads: Suppressor of Mothers against Decapentaplegic, MST1/2: mammalian STE20-like protein kinase 1/2, YAP: Yes-associated protein, TAZ: transcriptional coactivator with PDZ-binding motif, miR-21-5p: microRNA-21-5p, P38: p38 Mitogen-Activated Protein Kinase, VDR: vitamin D receptor, miR-132: microRNA-132, DCN: decorin, ZEB2: Zinc finger E-box binding homeobox 2, STAT3: Signal Transducer and Activator of Transcription 3, ERK: Extracellular Signal-Regulated Kinase, MMP-9 - Matrix Metalloproteinase 9, miR-126-3p: microRNA-126,-3p WNT: Wingless-related integration site, A2B: Adenosine A2B Receptor, MR: aldosterone mineralocorticoid receptor, CXCL10: C-X-C motif chemokine ligand 10, Pou4f1: POU Class 4 Homeobox 1, TCF: T-Cell Factor.

Initially, an epithelial response to inflammatory monocytes and macrophages occurs. Secondly, there is the production of inflammatory cytokines (notably TGF- β , IL-4, IL-10, IL-13, IL-17), chemokines (such as CCL2 and CXCL10), and growth factors (e.g., CTGF, PDGF, EGF, BMP-7) (Black et al., 2019). In the third stage, degradation (from extracellular matrix (ECM) proteins like MMPs, ADAMTs, collagen type III) and excessive production of ECM (from proteins such as α-SMA, TIMPs, fibronectin, collagen type I) occur within the renal interstitium (Wight and Potter-Perigo, 2011). As fibrosis progresses, the fourth stage is typified by a decrease of renal interstitial mesenchymal cells, coupled by cellular transformation into myofibroblasts. Finally, the fifth stage sees the narrowing of renal capillaries, leading to ischemia and anoxia, and capillary rarefication within the interstitial region (Figure 1A) (Yang et al., 2021; Wei et al., 2022).

The first and fourth stages of renal fibrosis introduce cells with the potential to differentiate into pathological myofibroblasts. The rise in active myofibroblast numbers within the interstitial space correlates positively with fibrosis severity (Sun et al., 2016). This review highlights myofibroblast transformation, with a focus on the cell of origin and the less-explored molecular pathways driving this change.

As the main producer of fibrotic ECM, myofibroblasts are distinguished from quiescent fibroblasts by elevated levels of alpha-smooth muscle actin (α-SMA) (Vierhout et al., 2021). Throughout the fibrotic process in the kidney, myofibroblasts generate collagens (type I, III, IV, V, and VI) and produce fibrillar matrix components such as fibronectins, fibrillins, and deposit connective matrix elastins and tenascins, as well as smaller molecules like proteoglycans and matricellular proteins such as MMPs and TIMPs (Klingberg et al., 2013). Protein matrices aid in tissue degradation, blood flow inhibition, and structural stiffening. Myofibroblast release of cytokines and chemokines like IL-1α, IL-1 β , IL-6, TNF-α, and CCL2 also exacerbate interstitial damage (Baum and Duffy, 2011; Vierhout et al., 2021). It's noteworthy that the process of renal fibrosis and the action of the myofibroblast vary across different types of renal insults and diseases, encompassing allograft rejection, glomerular nephritis, proteinuria, and tubulointerstitial disease (Mack and Yanagita, 2015).

While subject to controversy, fibroblasts, pericytes, endothelial cells, and macrophages collectively contribute significantly to myofibroblast formation in the renal interstitium during fibrosis. The ongoing debate over myofibroblast origin is fueled by differing methodologies used for cell fate tracing (Humphreys et al., 2010), and is further complicated by the impact of renal pathology on myofibroblast origin. LeBleu et al. reported that the contribution of epithelial-to-mesenchymal transition (EMT) to myofibroblasts is less than 5%, indicating a considerably lower role for this transition compared to demonstrated transformations of fibroblasts, pericytes, endothelial cells, and macrophages (Oldfield et al., 2001; Iwano et al., 2002; LeBleu et al., 2013; Falke et al., 2015). In several studies, renal perivascular fibroblasts have been shown to contribute to the myofibroblast pool during fibrosis, as much as 94% as determined by P0-Cre lineage-labelling (Asada et al., 2011). Pericytes, constituting approximately 2.5%–5% of all kidney cells also destabilize capillaries, leading to peritubular capillary rarefaction (Schrimpf and Duffield, 2011). In rat angiotensin II-induced kidney injury, pericyte transformation contributed to approximately 87% of myofibroblast formation (Faulkner et al., 2005). Conversely, in mice with unilateral ureteral obstruction (UUO), pericyte transformation accounted for either 6% or 0% of myofibroblast formation (LeBleu et al., 2013). Endothelial-to-mesenchymal transition (EndoMT) determined by double labelling with pan-endothelial marker, platelet endothelial cell adhesion molecule 1 (PECAM-1), αSMA, and *Tie2*-YFP lineage-traced cells in rodent models of kidney disease discovered that the EndoMT process contributed to ∼40% of all myofibroblasts (Zeisberg et al., 2008). Circulating monocyte-derived macrophages play a key role in renal injury, secreting pro-inflammatory cytokines and undergoing myofibroblast transformation. Studies on obstructive nephropathy reveal their contribution to the myofibroblast pool, ranging from 35% to as much as 90%, as observed through red fluorescent transgene tracing controlled by *Acta2* and identification using F4/80 and α-SMA markers (LeBleu et al., 2013; Vierhout et al., 2021).

Transforming growth factor beta (TGF- β ), a well-established profibrotic molecule, is the main driver that guides cellular transdifferentiation to form the myofibroblast (Yang et al., 1999; Iwano et al., 2002; Li and Bertram, 2010; Mariasegaram et al., 2010; Qin et al., 2011; Wu et al., 2013; Loeffler and Wolf, 2015; Wang et al., 2016; Humphreys, 2018). However, TGF-β has effects beyond fibrosis, and under certain circumstances, its inhibition can stimulate tumorigenesis (Garber, 2009). In fact, the use of TGF- β -neutralizing monoclonal anitbody, Fresolimumab, led to an increased rate of cutaneous squamous cell carcinoma development in patients, which promptly regressed upon antibody treatment cessation (Lacouture et al., 2015). Complicating its suitability as a drug target, TGF- β 's influence on active fibrogenic myofibroblast transdifferentition and proliferation is also cell-dependent and dose-dependent (Hathaway et al., 2015; Meng et al., 2016). Furthermore, receptor targeting by conditional blockade through the deletion of TGF-β type II receptor (TGF-IIR) on matrix-producing interstitial cells has failed to effectively reduce fibrosis severity in a model of mouse unilateral ureteral obstruction and in an aristolochic acid renal injury model suggesting the involvement of alternative signaling pathways (Neelisetty et al., 2015).

Canonical TGF- β signaling involves Smad2/3 phosphorylation and activation (Derynck and Zhang, 2003). The non-canonical TGF- β /Smad route typically activates ERK, the mitogen-activated protein kinase (MAPK), PI3K/Akt, and Rho GTPase downstream in the absence of Smad activity (Moustakas and Heldin, 2005). MicroRNA-mediated regulation (Bijkerk et al., 2016) and cross-talk with pathways such as Wnt/ β -catenin, Notch, Hedgehog, and NF-κB contribute to the complexity (Lamouille et al., 2014). Although blocking some well-known intermediaries partially inhibits renal interstitial fibrosis, compensatory activation often undermines lasting reparative effects (Maarouf et al., 2015).

TGF-β/Smad pathway targets remain ineffective or have unintended impacts in renal fibrosis (Klinkhammer et al., 2017). Emerging molecular targets beyond the canonical TGF- β axis show promise for fibrotic repair (Meng et al., 2016). This review, though non-exhaustive, highlights these targets and associated pathways, providing a foundation for understanding the dynamic nature of myofibroblast formation in kidney fibrosis (Duffield, 2014). It may reveal potential regenerative interventions to modulate non-essential pathways. Table 1 includes additional emerging targets beyond the reviewed molecules to capture a broader framework of these novel pathways.

**TABLE 1:** Emerging targets outside classical TGF-β signaling in renal fibrosis.

Target molecule		Reference	Direction favoring transdifferentiation	Associated pathway	Models
Fibroblasts - Demonstrated to contribute ∼94% of myofibroblast formation (Asada et al., 2011)					
Kif26b	Kinesin Family Member 26B	Yamamura et al. (2022)	Decrease	Increases NMHCII/Myh9	AN Kif26b het mice, primary renal fibroblasts
RALDH2	Retinaldehyde dehydrogenase 2	Nakamura et al. (2019)	Increase	Downstream of RALDH2 tubular cell/fibroblast crosstalk	IgA patients; AN, IRI mice
Nupr1	Nuclear protein 1	Zhou et al. (2021)	Increase	Drives TGF- β 1/Smad3 signaling	UUO Nupr1 KO mice, NRK-49F
Zeb2	Zinc finger E-box binding homeobox 2	Kumar et al. (2023)	Decrease	Smad/Wnt/AXIN2 downstream	Zeb2-cKO
FGF23	Bone-derived fibroblast growth factor 23	Smith et al. (2017)	Increase	Drives FGFR4 activation of TGF- β 1	UUO Sprague-Dawley primary kidney fibroblasts
WISP3	WNT1-inducible-signaling pathway protein 3	Yi et al. (2018)	Decrease	Induces Wnt-1/TGF- β signaling	UUO rat, NRK-49F cells
Pericytes - Demonstrated to contribute ∼ 0–87% of myofibroblast formation (Faulkner et al., 2005; LeBleu et al., 2013)					
FUT8	Fucosyltransferase 8	Wang et al. (2017)	Decrease	Inhibits TGF- β /Smad and PDGF/ERK pathways	IgAN patients, UUO mice
C5a	Complement component C5	Castellano et al. (2018)	Increase	Complement cascade activating TGF β	I/R swine, I/R C5aR1KO mice
Stat3	Signal transducer and activator of transcription 3	Ajay et al. (2022)	Increase	Induces Stat3 phosphorylation and nucear translocation	10T1/2 pericyte-like cells, Stat3KO mice
HKII	Hexokinase II	Chen et al. (2023)	Increase	Upregulates PI3K-Akt-mTOR glycolysis	PDGFR β +pericytes with HKII transfection
TLR-4	Toll-Like Receptor 4	Castellano et al. (2019)	Increase	Upregulates (LPS)-binding protein and TGF- β	LPS-induced AKI swine
Endothelial cells - Demonstrated to contribute ∼40% of myofibroblast formation (Zeisberg et al., 2008)					
C3a/C5a	Complement component C3a/C5a	Curci et al. (2014)	Increase	Activates Akt	I/R swine
SIRT3	SIRT (Sirtuin) 3	Lin et al. (2018)	Decrease	Activates Foxo3a-catalase pathway	Ang II renal fibrosis SIRT3 KO
Macrophages - Demonstrated to contribute ∼35%–90% of myofibroblast formation (LeBleu et al., 2013; Vierhout et al., 2021)					
Src	Steroid receptor coactivator	Tang et al. (2018)	Increase	Increases Smad3/TGF- β	UUO, Smad3 KO
GSDMD	Gasdermin D	Wang et al. (2022)	Decrease	Activates caspase-11/GSDMD-dependent NETs TGF- β /Smad3	UUO, Gsdmd KO mice
Jmjd3/IRF4	Jumonji domain-containing protein D3/Interferon regulatory factor 4	Liang et al. (2022)	Increase	Drives M2 macrophages to myofibroblasts	FA, UUO mice, IRF4 KO, Jmjd3f cKO mice
CD1d	Cluster of differentiation 1	Liu et al. (2021)	Decrease	Increases NKT cell/IL-4 signaling	FA, UUO mice, CD1d KO
FABP4	Fatty Acid-Binding Protein 4	Feng et al. (2020a)	Decrease	Reduces Saa1	UUO, IgAN patients, CKD patients
MR	Mineralocorticoid receptor	Qiang et al. (2022)	Decrease	ALD/MR/TGF- β 1 pathway	UUO
IRF-4	Interferon regulatory factor 4	Chen et al. (2021)	Increase	Increases CXCL16, IL-18, IL-6, TGF- β 1	FA IRF-4 KO mice

Emerging targets outside classical TGF-β, signaling in renal fibrosis. Table abbreviations - FA: Folic acid UUO: Unilateral Ureteral Obstruction AKI: Acute kidney injury NRK-49F: rat renal fibroblast cells MRPECs: mice renal peritubular endothelial cells IgAN: Immunoglobulin A nephropathy CKD: Chronic kidney disease KO: Knockout cKO: Conditional knockout I/R: Ischemia/Reperfusion AngII: Angiotensin II NETs: Neutrophil extracellular traps AN: adenine nephropathy.

### Fibroblast to myofibroblast

The yes-associated protein (YAP) signaling pathway has been shown in renal models to regulate fibrosis (An et al., 2022). YAP is part of the Hippo signaling pathway known to control cell growth, proliferation and organ size (Piccolo et al., 2014). It also plays a role in promoting fibroblast activation and myofibroblast differentiation induced by Smad/TGF- β signaling. Interestingly, the extent of TGF-β stimulation appears to dictate YAP’s function. Short exposures to TGF- β lead to YAP translocating into the nucleus, where it upregulates profibrotic and myofibroblast transdifferentiation-related genes. However, with prolonged and high-level TGF- β exposure, mammalian STE20-like protein kinase 1/2 (MST1/2) activation inhibits YAP translocation, effectively blocking a profibrotic response. This intricate regulatory mechanism involves MST1/2, acting as a negative regulator of YAP. Notably, knockout of *Mst1/2* in platelet-derived growth factor receptor-α (PDGFRα)+ specific cells leads to increased YAP expression, concomitant myofibroblast accumulation, and heightened renal fibrosis. Given that approximately 80% of PDGFRα+ or α-SMA + fibroblasts also happen to be YAP + cells, this signaling pathway emerges as a potentially dominant mechanism to drive the transition of fibroblasts to myofibroblasts (An et al., 2022).

Associated with YAP molecular signaling, another molecule emerges-transcriptional coactivator with PDZ-binding motif (TAZ), a transcriptional coactivator also within the Hippo pathway. Recent studies conducted in both mouse and human tissues have unveiled a notable pattern: following kidney transplant injury and in mouse models of UUO and ischemia-reperfusion injury (IRI) injury, the levels of YAP and TAZ in myofibroblasts surge dramatically. However, when myofibroblast YAP/TAZ is in short supply, fibrosis in unilateral ureteral obstruction-induced kidneys is diminished. Conversely, an excess of fibroblast YAP/TAZ intensifies fibrotic injury.

Furthermore, canonical transient receptor potential channel 6 (TRPC6), a nonselective cation channel, has been linked to TGF- β 1-induced fibroblast-to-myofibroblast transition in renal interstitial fibroblasts. TRPC6 expression is regulated by TGF- β 1 and is mediated through the p38/YAP pathway in renal interstitial fibroblast NRK-49F cells. NRK-49F *Trpc6* inhibition by siRNA or through pharmalogical intervention reduces expression levels of α-smooth muscle actin (α-SMA) and collagen I, both classical markers of myofibroblasts. Jiang et al. demonstrate that TGF- β 1 exposure causes phosphorylation of p38 and Yes-associated protein, leading to translocation into the nuclei. Inhibition p38/YAP phosphorylation decreases TRPC6 and α-SMA expression, indicating a key role for TRPC6 in TGF- β 1 stimulated fibroblast to myofibroblast transition that is also downstream of the p38/YAP pathway (Jiang et al., 2022).

Lastly, TGF- β -induced fibroblast-to-myofibroblast transition is associated with an upregulation of microRNA-21-5p (miR-21-5p). MiR-21-5p has several well-established target genes that are involved in the regulation of fibrosis. These targets include PTEN (Phosphatase and Tensin Homolog), PDCD4 (Programmed Cell Death 4), SPRY1 (Sprouty Homolog 1), and PPARα which regulates fatty acid oxidation in mitochondria. (Ren and Duffield, 2013). By downregulating these anti-fibrotic genes, miR-21-5p promotes profibrotic responses. Anti-fibrotic effects of melatonin treatment was demonstrated to reduce renal fibrosis, partially by downregulating α-SMA, Col1a1, fibronectin, and miR-21-5p. While, the beneficial effects of melatonin treatment are reversed by agomirs of miR-21-5p in UUO mouse models (Yang et al., 2013; Li et al., 2020).

### Pericyte to myofibroblast

Studies utilizing pathway and gene expression analysis implicate miR-132 in pericyte transformation under TGF- β /Smad2/Smad3 control, networking with both cell proliferation-related signaling, STAT3/ERK and Foxo3/p300. The role of miR-132 becomes particularly evident in FoxD1-GC; Z/Red-mice subjected to unilateral ureteral obstruction, where pericytes undergo a remarkable 21-fold upregulation of miR-132 during the transition from pericytes to myofibroblasts. This surge in miR-132 coincides with heightened myofibroblast transformation and an increase in collagen deposition, ultimately contributing to kidney fibrosis. When miR-132 is deliberately silenced, not only does collagen deposition decrease, but myofibroblast proliferation is attenuated. However, in the context of TGF- β control over pericyte-to-myofibroblast transformation, it's worth noting that while TGF-β stimulation in pericyte cultures typically triggers myofibroblast differentiation without significant proliferation, this is not the case downstream of miR-132 activation (Ren and Duffield, 2013), suggesting a more intricate regulatory role for this microRNA in the transformation process.

Zinc finger E-box binding homeobox 2 (ZEB2), a promoter region with binding sites for FOXD1, exerts inhibitory control over both transcription factor, Zeb2 and decorin (DCN). An intriguing aspect of DCN, a proteoglycan, is that it sequesters TGF- β , thereby neutralizing its binding capability and subsequent signaling cascade and it acts as an antagonist to SMAD signaling, particularly during nephron development. Therefore, it is plausible that Zeb2 may ultimately regulate upstream of SMAD which would explain why when inhibited in Zeb2 cKO mice there is differentiation of Foxd1-positive pericytes into myofibroblasts, ultimately contributing to the progression of renal fibrosis (Fetting et al., 2014; Kumar et al., 2023).

In a comprehensive study investigating the impact of inflammation-induced upregulation of vitamin D receptors (VDR) in the kidney, a significant link was established between VDR activators and the conversion of pericytes into myofibroblasts. This study examined the influence of VDR activators in the context of hypoxia or in the presence of TGF- β 1, both of which initiate pericyte transformation in a HIF-1α-dependent manner, primarily through the Smad2 signaling pathway. Notably, the introduction of paricalcitol, a ligand targeting VDRs, resulted in a marked inhibition of pericyte transformation. Intervention coincided with a concurrent reduction in the expression of key markers, TGF- β 1, α-SMA, and PDGFR β in pericytes (El-Atifi et al., 2015). This study not only suggests a VDR link to pericyte transdifferentiation but also highlights the pivotal role of hypoxia in orchestrating HIF-1α, which integrates multiple signaling networks such as the TGF- β /Smad, Notch, and NF-κB pathways in the induction of kidney fibrosis (Liu et al., 2017; Lim et al., 2021).

### Endothelial to mesenchymal transition

In UUO mice induced for kidney fibrosis, matrix metalloproteinase 9 (MMP-9) knockouts have reduced EndoMT, evidenced by decreased histological VE-cadherin and α-SMA colocalization compared to wildtype controls. Primary endothelial cells (MRPECs) treated with recombinant TGF- β 1 and MMP-9 inhibitor also demonstrated the same reduction in EndoMT. These changes correlated with a decrease in Notch activation and its downstream reduction of transcription factor, Hey-1, indicating a MMP-9 dependent -Notch regulation of myofibroblast formation (Zhao et al., 2017).

While ERK1/2 signaling is crucial in development and homeostasis, modest pharmacological intervention to prevent inhibition of ERK1/2 signaling may halt endothelial-to-myofibroblast transformation (Cao et al., 2019). Mice lacking global ERK1 and containing endothelial-specific ERK2 (Erk1^−/−^ Erk2^iEC−/−^) by two and 3 weeks of age undergo myofibroblast transition of endothelial cells as indicated by VE cadherin + αSMA + expression in the kidney. By 5 weeks of age, these mice succumb to organ failure, primarily a result of fibrosis of the heart. An increase of EndoMT was replicated through siRNA inhibition of *ERK1/2* signaling of human endothelial cells (HUVECs) when compared to wildtype controls. RNA Seq analysis of these siRNA treated cells and scrambled controls indicated a gene hub that consisted of a *TGF*- β *2* gene driver with downstream regulation of ECM genes *COL25A1*, *COL5A1*, and *CDH2* vasoactive genes *EDN1* and *NOS3*, and fenestration gene, *PVLAP* (Ricard et al., 2019).

MiRNA-driven myofibroblast transformation is evident in endothelial cells, as indicated by a study highlighting the regulatory role of miR-126-3p. Endothelial derived myofibroblasts were traced by YFP under a *Cdh5* promoter in mice which were induced to kidney fibrosis by unilateral ureteral obstruction. Five days post-operation, YFP + cells differentiated within the glomeruli, capillaries, and blood vessel intima, and represented 9% of αSMA + cells. Examination via RT-qPCR and *in situ* hybridization of kidney sections revealed a significant downregulation of miR-126-3p in fibrotic mouse kidneys compared to healthy kidneys. This downregulation was also observed in fibrotic human kidneys compared to normal kidneys. Diminished levels of miR-126-3p are evident in diverse injury contexts, including human renal IRI and myocardial infarction, indicating its prospective utility as a valuable molecular biomarker for disease monitoring and a potential target for intervention (Jordan et al., 2021).

### Macrophage to myofibroblast

Studies utilizing chromatin immunoprecipitation (ChIP) techniques have identified POU Class 4 Homeobox 1 (Pou4f1) as a downstream target of Smad3 and a regulator of macrophage-to-myofibroblast transition (MMT). Microarray analysis further revealed Pou4f1 as a pivotal node in a fibrogenic gene network that promotes TGF- β 1/Smad3-driven MMT in bone-marrow derived macrophages, suggesting Pou4f1 as a potentially viable therapeutic target. Macrophage-specific inhibition of Pou4f1 signaling, both *in vitro* and *in vivo* using UUO and IRI mouse models of kidney fibrosis, effectively impedes macrophage transition to myofibroblasts and halts fibrosis progression. Notably, elevated Pou4f1 levels also show a strong correlation with increased renal injury associated with fibrosis in human diseases (Tang et al., 2020).

The TGF- β /Smad signaling pathway dynamically engages with the Wnt/ β -catenin pathway. A fundamental part of the canonical Wnt signaling cascade is mediated by β-catenin and T-cell factor (TCF). Interventions that hinder β -catenin’s interaction with TCF not only reduce MMT but also steer β -catenin towards interacting with Foxo1 during the progression of renal fibrosis (Vierhout et al., 2021). Intriguingly, the non-canonical Wnt signaling pathway can be set into motion through TGF- β 1 induction, where Wnt5a operates independently by elevating YAP/TAZ levels. This elevation then triggers the induction of macrophage M2 polarization, driving forward myofibroblast transformation and the fibrotic response (An et al., 2022).

In a concurrent signaling cascade, the A2B adenosine receptor, notable for its ability to initiate G protein signaling that sets in motion a multitude of intracellular processes, such as alterations in gene expression, ion channel activity, and metabolism, showcases the potential to amplify the TGF- β pathway. This augmentation, in turn, is implicated in an escalation of renal fibrosis by instigating MMT. Experimental treatments involving the use of an A2B adenosine receptor antagonist in diabetic rats revealed a notable decrease in glomerulosclerosis, reduced levels of collagen and α-SMA, and a significant reduction in MMT, indicating a promising approach for mitigating renal fibrosis (Cardenas et al., 2013; Torres et al., 2020).

Another receptor pathway-the aldosterone mineralocorticoid receptor (MR) has been shown to aide the transition of M1 macrophages to myofibroblasts upon stimulation of TGF- β . Inhibition of this pathway using the MR blocker, esaxerenone, has been demonstrated to mitigate the fibrotic phenotype and reduce macrophage-myofibroblast transition both in *in vivo* and *in vitro* settings (Qiang et al., 2022).

Lastly, analyzing the transcriptome of macrophages treated with TGF- β revealed the involvement of CXCL10 in the signaling pathway. Notably, in UUO mice, there exists a noteworthy positive correlation between the accumulation of MMT and increased levels of CXCL10. Interventions targeting CXCL10, whether through siRNA knockdown techniques, or chemical inhibition, resulted in a reduction of MMT in mouse models of kidney fibrosis, underlining the dynamic impact of CXCL10 (Feng et al., 2020a). It is worth mentioning that CXCL10 exhibits a dual molecular potential, sometimes acting as an antifibrotic agent, depending on the specific fibrotic context (Tager et al., 2004; Roman and Mutsaers, 2018; Feng et al., 2020b).

## Discussion

It is important to recognize that often the mechanism driving alterations in myofibroblast cells involves paracrine cross-talk. Tubular cells, when damaged during renal fibrosis, transition to a secretory phenotype and generate fibrogenic agents such as sonic hedgehog (Shh), Wnt ligands, and TGF- β , as well as IL-6, monocyte chemotactic protein-1, TNF-α, and other inflammatory cytokines. These molecules contribute to the activation and transdifferentiation of myofibroblasts. Often, this paracrine interaction is facilitated through the production and transmission of mRNA exosomes containing factors such as TGF- β , delivered to neighboring fibroblasts (Mack and Yanagita, 2015). An example of paracrine communication at the cellular level can be observed with putative endothelial progenitor cells (pEPCs) or their microvesicles, which are believed to secrete factors that inhibit TGF- β regulation, effectively reducing pericyte-to-myofibroblast transition. Following pEPC treatment, UUO-injured mice not only exhibit reduced expression of α-SMA and Collagen IV but also experience decreased myofibroblast accumulation and mitigation of renal fibrosis (Schrimpf and Duffield, 2011; Smith et al., 2012; Yang et al., 2019).

Since the hallmark of successful renal fibrosis resolution in disease models includes a decrease in myofibroblasts (Sun and Kisseleva, 2015), comprehending myofibroblast generation dynamics is key in identifying therapeutic targets in renal fibrotic diseases. In order to realistically carry out myofibroblast transdifferentiation inhibition it is reasonable to either alter the secretory phenotype of kidney resident cells or to block circulating recruitment into the renal interstitium of bone marrow derived macrophages fated for MMT (Kok et al., 2014; Meng et al., 2014; Tampe and Zeisberg, 2014). Targeting the TGF- β canonical or non-canonical signaling pathways within the renal interstitium could be achieved through a hydrogel depot containing inhibitors such as microRNA-21-5p/miR-132 antagonists and CXCL10 inhibitors (Adler et al., 2010; Qin et al., 2011) and may present a feasible method to inhibit local activation and proliferation of myofibroblasts.

Expanding the search for targets outside of the classical TGF- β /Smad pathway is crucial for advancing antifibrotic therapies. Currently, ACE-Is and ARBs dominate therapeutic options for renal fibrosis (Liu and Zhuang, 2019). While therapies addressing leukocyte recruitment, soluble factors, and matrix protein production are valuable, strategically targeting myofibroblasts at the developmental stage emerges as a promising approach to control excessive extracellular matrix deposition and impede fibrosis progression (Tampe and Zeisberg, 2014).

## Figures and Tables

**Table:** 

**A2B**	Adenosine A2B Receptor
**ACE-Is**	Angiotensin-Converting Enzyme Inhibitors
**ADAMTs**	A Disintegrin And Metalloproteinase with Thrombospondin Motifs
**Akt**	Protein Kinase B
**α-SMA**	Alpha-Smooth Muscle Actin
β **-catenin**	Beta-Catenin
**BMP-7**	Bone Morphogenetic Protein 7
**CDH2**	Cadherin 2
**Cdh5**	Cadherin 5
**ChIP**	Chromatin Immunoprecipitation
**COL25A1**	Collagen Type XXV Alpha 1 Chain
**COL5A1**	Collagen Type V Alpha 1 Chain
**CTGF**	Connective Tissue Growth Factor
**CXCL10**	C-X-C Motif Chemokine Ligand 10
**DCN**	Decorin
**ECM**	Extracellular Matrix
**EMT**	Epithelial-to-Mesenchymal Transition
**EndoMT**	Endothelial-to-Mesenchymal Transition
**ERK**	Extracellular Signal-Regulated Kinase
**FOXD1**	Forkhead Box D1
**Foxd1**	Forkhead Box D1
**Foxo3**	Forkhead Box O3
**GC**	Glucocorticoid
**G protein**	Guanine Nucleotide-Binding Protein
**HIF-1α**	Hypoxia-Inducible Factor 1 Alpha
**Hippo**	Hippo Signaling Pathway
**HUVECs**	Human Umbilical Vein Endothelial Cells
**IL**	Interleukin
**IL-6**	Interleukin-6
**IRI**	Ischemia-Reperfusion Injury
**MAPK**	Mitogen-Activated Protein Kinase
**MMP-9**	Matrix Metalloproteinase 9
**MMPs**	Matrix Metalloproteinases
**MMT**	Macrophage-to-Myofibroblast Transition
**mRNA**	Messenger RNA
**MR**	Mineralocorticoid Receptor
**MRPECs**	Primary Endothelial Cells
**MST1/2**	Mammalian STE20-like Protein Kinase 1/2
**NF-κB**	Nuclear Factor Kappa B
**NOS3**	Nitric Oxide Synthase 3
**NRK-49F**	Normal Rat Kidney Fibroblast
**P38**	p38 Mitogen-Activated Protein Kinase
**PDGF**	Platelet-Derived Growth Factor
**PDGFRα**	Platelet-Derived Growth Factor Receptor-α
**PDGFR** β	Platelet-Derived Growth Factor Receptor Beta
**PDCD4**	Programmed Cell Death 4
**PECAM-1**	Platelet Endothelial Cell Adhesion Molecule 1
**PI3K**	Phosphoinositide 3-Kinase
**PPARα**	Peroxisome Proliferator-Activated Receptor Alpha
**Pou4f1**	POU Class 4 Homeobox 1
**pEPCs**	Putative Endothelial Progenitor Cells
**PTEN**	Phosphatase and Tensin Homolog
**PVLAP**	Plasmalemma Vesicle-Associated Protein
**Rho GTPase**	Rho Guanosine Triphosphatase
**RNA Seq**	RNA Sequencing
**Shh**	Sonic Hedgehog
**siRNA**	Small Interfering RNA
**SMAD**	Mothers Against Decapentaplegic Homolog
**SPRY1**	Sprouty Homolog 1
**STAT3**	Signal Transducer and Activator of Transcription 3
**TAZ**	Transcriptional Coactivator with PDZ-Binding Motif
**TCF**	T-Cell Factor
**TGF-** β	Transforming Growth Factor Beta
**TGF-** β **1**	Transforming Growth Factor Beta 1
**TGF IIR**	TGF- β Type II Receptor
**TIMPs**	Tissue Inhibitors of Metalloproteinases
**TRPC6**	Transient Receptor Potential Channel 6
**UUO**	Unilateral Ureteral Obstruction
**VE-cadherin**	Vascular Endothelial Cadherin
**Wnt**	Wingless/Integrated
**YAP**	Yes-Associated Protein
**YFP**	Yellow Fluorescent Protein
**ZEB2**	Zinc Finger E-Box Binding Homeobox 2
**Z/Red**	Zeigler/Red Fluorescent Protein

## References

[B1] Adler S. G. Schwartz S. Williams M. E. Arauz-Pacheco C. Bolton W. K. Lee T. (2010). Phase 1 study of anti-CTGF monoclonal antibody in patients with diabetes and microalbuminuria. Clin. J. Am. Soc. Nephrol. 5 (8), 1420–1428. 10.2215/CJN.09321209 20522536 PMC2924405

[B2] Ajay A. K. Zhao L. Vig S. Fujiwara M. Thakurela S. Jadhav S. (2022). Deletion of STAT3 from Foxd1 cell population protects mice from kidney fibrosis by inhibiting pericytes trans-differentiation and migration. Cell Rep. 38 (10), 110473. 10.1016/j.celrep.2022.110473 35263586 PMC10027389

[B3] An Y. Ren Y. Wang J. Zang J. Gao M. Wang H. (2022). MST1/2 in PDGFRα(+) cells negatively regulates TGF-β-induced myofibroblast accumulation in renal fibrosis. Am. J. Physiol. Ren. Physiol. 322 (5), F512–f526. 10.1152/ajprenal.00367.2021 35253468

[B4] Asada N. Takase M. Nakamura J. Oguchi A. Asada M. Suzuki N. (2011). Dysfunction of fibroblasts of extrarenal origin underlies renal fibrosis and renal anemia in mice. J. Clin. Investig. 121 (10), 3981–3990. 10.1172/JCI57301 21911936 PMC3195468

[B5] Baum J. Duffy H. S. (2011). Fibroblasts and myofibroblasts: what are we talking about? J. Cardiovasc Pharmacol. 57 (4), 376–379. 10.1097/FJC.0b013e3182116e39 21297493 PMC3077448

[B6] Bijkerk R. de Bruin R. G. van Solingen C. van Gils J. M. Duijs J. M. van der Veer E. P. (2016). Silencing of microRNA-132 reduces renal fibrosis by selectively inhibiting myofibroblast proliferation. Kidney Int. 89 (6), 1268–1280. 10.1016/j.kint.2016.01.029 27165825

[B7] Black L. M. Lever J. M. Agarwal A. (2019). "Renal inflammation and fibrosis: a double-edged sword. J. Histochem Cytochem 67 (9), 663–681. 10.1369/0022155419852932 31116067 PMC6713973

[B8] Cao L. Yuan X. Bao F. Lv W. He Z. Tang J. (2019). Downregulation of HSPA2 inhibits proliferation via ERK1/2 pathway and endoplasmic reticular stress in lung adenocarcinoma. Ann. Transl. Med. 7 (20), 540. 10.21037/atm.2019.10.16 31807522 PMC6861743

[B9] Cardenas A. Toledo C. Oyarzun C. Sepulveda A. Quezada C. Guillen-Gomez E. (2013). Adenosine A(2B) receptor-mediated VEGF induction promotes diabetic glomerulopathy. Lab. Investig. 93 (1), 135–144. 10.1038/labinvest.2012.143 23069939

[B10] Castellano G. Franzin R. Stasi A. Divella C. Sallustio F. Pontrelli P. (2018). Complement activation during ischemia/reperfusion injury induces pericyte-to-myofibroblast transdifferentiation regulating peritubular capillary lumen reduction through pERK signaling. Front. Immunol. 9, 1002. 10.3389/fimmu.2018.01002 29875766 PMC5974049

[B11] Castellano G. Stasi A. Franzin R. Sallustio F. Divella C. Spinelli A. (2019). LPS-binding protein modulates acute renal fibrosis by inducing pericyte-to-myofibroblast trans-differentiation through TLR-4 signaling. Int. J. Mol. Sci. 20 (15), 3682. 10.3390/ijms20153682 31357597 PMC6696277

[B12] Chen L. Li X. Deng Y. Chen J. Huang M. Zhu F. (2023). The PI3K-Akt-mTOR pathway mediates renal pericyte-myofibroblast transition by enhancing glycolysis through HKII. J. Transl. Med. 21 (1), 323. 10.1186/s12967-023-04167-7 37179292 PMC10182641

[B13] Chen M. Wen X. Gao Y. Liu B. Zhong C. Nie J. (2021). IRF-4 deficiency reduces inflammation and kidney fibrosis after folic acid-induced acute kidney injury. Int. Immunopharmacol. 100, 108142. 10.1016/j.intimp.2021.108142 34555644

[B14] Curci C. Castellano G. Stasi A. Divella C. Loverre A. Gigante M. (2014). Endothelial-to-mesenchymal transition and renal fibrosis in ischaemia/reperfusion injury are mediated by complement anaphylatoxins and Akt pathway. Nephrol. Dial. Transpl. 29 (4), 799–808. 10.1093/ndt/gft516 24463188

[B15] Derynck R. Zhang Y. E. (2003). Smad-dependent and Smad-independent pathways in TGF-beta family signalling. Nature 425 (6958), 577–584. 10.1038/nature02006 14534577

[B16] Duffield J. S. (2014). Cellular and molecular mechanisms in kidney fibrosis. J. Clin. Investig. 124 (6), 2299–2306. 10.1172/JCI72267 24892703 PMC4038570

[B17] El-Atifi M. Dreyfus M. Berger F. Wion D. (2015). Expression of CYP2R1 and VDR in human brain pericytes: the neurovascular vitamin D autocrine/paracrine model. Neuroreport 26 (5), 245–248. 10.1097/WNR.0000000000000328 25730676

[B18] Falke L. L. Gholizadeh S. Goldschmeding R. Kok R. J. Nguyen T. Q. (2015). Diverse origins of the myofibroblast—implications for kidney fibrosis. Nat. Rev. Nephrol. 11 (4), 233–244. 10.1038/nrneph.2014.246 25584804

[B19] Faulkner J. L. Szcykalski L. M. Springer F. Barnes J. L. (2005). Origin of interstitial fibroblasts in an accelerated model of angiotensin II-induced renal fibrosis. Am. J. Pathol. 167 (5), 1193–1205. 10.1016/S0002-9440(10)61208-4 16251405 PMC1603794

[B20] Feng Y. Guo F. Mai H. Liu J. Xia Z. Zhu G. (2020a). Pterostilbene, a bioactive component of blueberries, alleviates renal interstitial fibrosis by inhibiting macrophage-myofibroblast transition. Am. J. Chin. Med. 48 (7), 1715–1729. 10.1142/S0192415X20500858 33148003

[B21] Feng Y. Guo F. Xia Z. Liu J. Mai H. Liang Y. (2020b). Inhibition of fatty acid-binding protein 4 attenuated kidney fibrosis by mediating macrophage-to-myofibroblast transition. Front. Immunol. 11, 566535. 10.3389/fimmu.2020.566535 33101287 PMC7554244

[B22] Fetting J. L. Guay J. A. Karolak M. J. Iozzo R. V. Adams D. C. Maridas D. E. (2014). FOXD1 promotes nephron progenitor differentiation by repressing decorin in the embryonic kidney. Development 141 (1), 17–27. 10.1242/dev.089078 24284212 PMC3865747

[B23] Garber K. (2009). Companies waver in efforts to target transforming growth factor beta in cancer. J. Natl. Cancer Inst. 101 (24), 1664–1667. 10.1093/jnci/djp462 19933941

[B24] Hathaway C. K. Gasim A. M. Grant R. Chang A. S. Kim H. S. Madden V. J. (2015). Low TGFβ1 expression prevents and high expression exacerbates diabetic nephropathy in mice. Proc. Natl. Acad. Sci. U. S. A. 112 (18), 5815–5820. 10.1073/pnas.1504777112 25902541 PMC4426439

[B25] Humphreys B. D. (2018). Mechanisms of renal fibrosis. Annu. Rev. Physiol. 80, 309–326. 10.1146/annurev-physiol-022516-034227 29068765

[B26] Humphreys B. D. Lin S. L. Kobayashi A. Hudson T. E. Nowlin B. T. Bonventre J. V. (2010). Fate tracing reveals the pericyte and not epithelial origin of myofibroblasts in kidney fibrosis. Am. J. Pathol. 176 (1), 85–97. 10.2353/ajpath.2010.090517 20008127 PMC2797872

[B27] Iwano M. Plieth D. Danoff T. M. Xue C. Okada H. Neilson E. G. (2002). Evidence that fibroblasts derive from epithelium during tissue fibrosis. J. Clin. Investig. 110 (3), 341–350. 10.1172/JCI15518 12163453 PMC151091

[B28] Jiang S. Gu L. Hu Y. Ren Y. Yang Z. Chai C. (2022). Inhibition of TRPC6 suppressed TGFβ-induced fibroblast-myofibroblast transdifferentiation in renal interstitial NRK-49F cells. Exp. Cell Res. 421 (1), 113374. 10.1016/j.yexcr.2022.113374 36206825

[B29] Jordan N. P. Tingle S. J. Shuttleworth V. G. Cooke K. Redgrave R. E. Singh E. (2021). MiR-126-3p is dynamically regulated in endothelial-to-mesenchymal transition during fibrosis. Int. J. Mol. Sci. 22 (16), 8629. 10.3390/ijms22168629 34445337 PMC8395326

[B30] Klingberg F. Hinz B. White E. S. (2013). The myofibroblast matrix: implications for tissue repair and fibrosis. J. Pathol. 229 (2), 298–309. 10.1002/path.4104 22996908 PMC4005341

[B31] Klinkhammer B. M. Goldschmeding R. Floege J. Boor P. (2017). Treatment of renal fibrosis-turning challenges into opportunities. Adv. Chronic Kidney Dis. 24 (2), 117–129. 10.1053/j.ackd.2016.11.002 28284377

[B32] Kok H. M. Falke L. L. Goldschmeding R. Nguyen T. Q. (2014). Targeting CTGF, EGF and PDGF pathways to prevent progression of kidney disease. Nat. Rev. Nephrol. 10 (12), 700–711. 10.1038/nrneph.2014.184 25311535

[B33] Kumar S. Fan X. Rasouly H. M. Sharma R. Salant D. J. Lu W. (2023). ZEB2 controls kidney stromal progenitor differentiation and inhibits abnormal myofibroblast expansion and kidney fibrosis. JCI Insight 8 (1), e158418. 10.1172/jci.insight.158418 36445780 PMC9870089

[B34] Lacouture M. E. Morris J. C. Lawrence D. P. Tan A. R. Olencki T. E. Shapiro G. I. (2015). Cutaneous keratoacanthomas/squamous cell carcinomas associated with neutralization of transforming growth factor β by the monoclonal antibody fresolimumab (GC1008). Cancer Immunol. Immunother. 64 (4), 437–446. 10.1007/s00262-015-1653-0 25579378 PMC6730642

[B35] Lamouille S. Xu J. Derynck R. (2014). Molecular mechanisms of epithelial-mesenchymal transition. Nat. Rev. Mol. Cell Biol. 15 (3), 178–196. 10.1038/nrm3758 24556840 PMC4240281

[B36] LeBleu V. S. Taduri G. O'Connell J. Teng Y. Cooke V. G. Woda C. (2013). Origin and function of myofibroblasts in kidney fibrosis. Nat. Med. 19 (8), 1047–1053. 10.1038/nm.3218 23817022 PMC4067127

[B37] Li J. Bertram J. F. (2010). Review: endothelial-myofibroblast transition, a new player in diabetic renal fibrosis. Nephrol. Carlt. 15 (5), 507–512. 10.1111/j.1440-1797.2010.01319.x 20649869

[B38] Li N. Wang Z. Gao F. Lei Y. Li Z. (2020). Melatonin ameliorates renal fibroblast-myofibroblast transdifferentiation and renal fibrosis through miR-21-5p regulation. J. Cell Mol. Med. 24 (10), 5615–5628. 10.1111/jcmm.15221 32243691 PMC7214152

[B39] Liang H. Liu B. Gao Y. Nie J. Feng S. Yu W. (2022). Jmjd3/IRF4 axis aggravates myeloid fibroblast activation and m2 macrophage to myofibroblast transition in renal fibrosis. Front. Immunol. 13, 978262. 10.3389/fimmu.2022.978262 36159833 PMC9494509

[B40] Lim J. H. Yook J. M. Oh S. H. Jeon S. J. Noh H. W. Jung H. Y. (2021). Paricalcitol improves hypoxia-induced and TGF-β1-induced injury in kidney pericytes. Int. J. Mol. Sci. 22 (18), 9751. 10.3390/ijms22189751 34575914 PMC8472327

[B41] Lin J. R. Zheng Y. J. Zhang Z. B. Shen W. L. Li X. D. Wei T. (2018). Suppression of endothelial-to-mesenchymal transition by SIRT (sirtuin) 3 alleviated the development of hypertensive renal injury. Hypertension 72 (2), 350–360. 10.1161/HYPERTENSIONAHA.118.10482 29915018

[B42] Liu B. Jiang J. Liang H. Xiao P. Lai X. Nie J. (2021). Natural killer T cell/IL-4 signaling promotes bone marrow-derived fibroblast activation and M2 macrophage-to-myofibroblast transition in renal fibrosis. Int. Immunopharmacol. 98, 107907. 10.1016/j.intimp.2021.107907 34243040

[B43] Liu F. Zhuang S. (2019). New therapies for the treatment of renal fibrosis. Adv. Exp. Med. Biol. 1165, 625–659. 10.1007/978-981-13-8871-2_31 31399988

[B44] Liu M. Ning X. Li R. Yang Z. Yang X. Sun S. (2017). Signalling pathways involved in hypoxia-induced renal fibrosis. J. Cell Mol. Med. 21 (7), 1248–1259. 10.1111/jcmm.13060 28097825 PMC5487923

[B45] Loeffler I. Wolf G. (2015). Epithelial-to-Mesenchymal transition in diabetic nephropathy: fact or fiction? Cells 4 (4), 631–652. 10.3390/cells4040631 26473930 PMC4695850

[B46] Maarouf O. H. Ikeda Y. Humphreys B. D. (2015). Wnt signaling in kidney tubulointerstitium during disease. Histol. Histopathol. 30 (2), 163–171. 10.14670/HH-30.163 25297005

[B47] Mack M. Yanagita M. (2015). Origin of myofibroblasts and cellular events triggering fibrosis. Kidney Int. 87 (2), 297–307. 10.1038/ki.2014.287 25162398

[B48] Mariasegaram M. Tesch G. H. Verhardt S. Hurst L. Lan H. Y. Nikolic-Paterson D. J. (2010). Lefty antagonises TGF-beta1 induced epithelial-mesenchymal transition in tubular epithelial cells. Biochem. Biophys. Res. Commun. 393 (4), 855–859. 10.1016/j.bbrc.2010.02.098 20171171

[B49] Meng X. M. Nikolic-Paterson D. J. Lan H. Y. (2014). Inflammatory processes in renal fibrosis. Nat. Rev. Nephrol. 10 (9), 493–503. 10.1038/nrneph.2014.114 24981817

[B50] Meng X. M. Nikolic-Paterson D. J. Lan H. Y. (2016). TGF-β: the master regulator of fibrosis. Nat. Rev. Nephrol. 12 (6), 325–338. 10.1038/nrneph.2016.48 27108839

[B51] Moustakas A. Heldin C. H. (2005). Non-Smad TGF-beta signals. J. Cell Sci. 118 (Pt 16), 3573–3584. 10.1242/jcs.02554 16105881

[B52] Nakamura J. Sato Y. Kitai Y. Wajima S. Yamamoto S. Oguchi A. (2019). Myofibroblasts acquire retinoic acid-producing ability during fibroblast-to-myofibroblast transition following kidney injury. Kidney Int. 95 (3), 526–539. 10.1016/j.kint.2018.10.017 30661714

[B53] Neelisetty S. Alford C. Reynolds K. Woodbury L. Nlandu-Khodo S. Yang H. (2015). Renal fibrosis is not reduced by blocking transforming growth factor-β signaling in matrix-producing interstitial cells. Kidney Int. 88 (3), 503–514. 10.1038/ki.2015.51 25760325 PMC4556568

[B54] Oldfield M. D. Bach L. A. Forbes J. M. Nikolic-Paterson D. McRobert A. Thallas V. (2001). Advanced glycation end products cause epithelial-myofibroblast transdifferentiation via the receptor for advanced glycation end products (RAGE). J. Clin. Investig. 108 (12), 1853–1863. 10.1172/JCI11951 11748269 PMC209461

[B55] Piccolo S. Dupont S. Cordenonsi M. (2014). The biology of YAP/TAZ: hippo signaling and beyond. Physiol. Rev. 94 (4), 1287–1312. 10.1152/physrev.00005.2014 25287865

[B56] Qiang P. Hao J. Yang F. Han Y. Chang Y. Xian Y. (2022). Esaxerenone inhibits the macrophage-to-myofibroblast transition through mineralocorticoid receptor/TGF-β1 pathway in mice induced with aldosterone. Front. Immunol. 13, 948658. 10.3389/fimmu.2022.948658 36148244 PMC9485811

[B57] Qin W. Chung A. C. Huang X. R. Meng X. M. Hui D. S. Yu C. M. (2011). TGF-β/Smad3 signaling promotes renal fibrosis by inhibiting miR-29. J. Am. Soc. Nephrol. 22 (8), 1462–1474. 10.1681/ASN.2010121308 21784902 PMC3148701

[B58] Ren S. Duffield J. S. (2013). Pericytes in kidney fibrosis. Curr. Opin. Nephrol. Hypertens. 22 (4), 471–480. 10.1097/MNH.0b013e328362485e 23722183

[B59] Ricard N. Scott R. P. Booth C. J. Velazquez H. Cilfone N. A. Baylon J. L. (2019). Endothelial ERK1/2 signaling maintains integrity of the quiescent endothelium. J. Exp. Med. 216 (8), 1874–1890. 10.1084/jem.20182151 31196980 PMC6683988

[B60] Roman J. Mutsaers S. E. (2018). Epigenetic control of CXCL10: regulating the counterregulator in idiopathic pulmonary fibrosis. Am. J. Respir. Cell Mol. Biol. 58 (4), 419–420. 10.1165/rcmb.2017-0389ED 29717923

[B61] Schrimpf C. Duffield J. S. (2011). Mechanisms of fibrosis: the role of the pericyte. Curr. Opin. Nephrol. Hypertens. 20 (3), 297–305. 10.1097/MNH.0b013e328344c3d4 21422927

[B62] Smith E. R. Holt S. G. Hewitson T. D. (2017). FGF23 activates injury-primed renal fibroblasts via FGFR4-dependent signalling and enhancement of TGF-β autoinduction. Int. J. Biochem. Cell Biol. 92, 63–78. 10.1016/j.biocel.2017.09.009 28919046

[B63] Smith S. W. Schrimpf C. Parekh D. J. Venkatachalam M. Duffield J. S. (2012). Kidney pericytes: a novel therapeutic target in interstitial fibrosis. Histol. Histopathol. 27 (12), 1503–1514. 10.14670/HH-27.1503 23059881

[B64] Sun M. Kisseleva T. (2015). Reversibility of liver fibrosis. Clin. Res. Hepatol. Gastroenterol. 39 (Suppl. 1), S60–S63. 10.1016/j.clinre.2015.06.015 26206574 PMC4636085

[B65] Sun Y. B. Qu X. Caruana G. Li J. (2016). The origin of renal fibroblasts/myofibroblasts and the signals that trigger fibrosis. Differentiation 92 (3), 102–107. 10.1016/j.diff.2016.05.008 27262400

[B66] Tager A. M. Kradin R. L. LaCamera P. Bercury S. D. Campanella G. S. Leary C. P. (2004). Inhibition of pulmonary fibrosis by the chemokine IP-10/CXCL10. Am. J. Respir. Cell Mol. Biol. 31 (4), 395–404. 10.1165/rcmb.2004-0175OC 15205180

[B67] Tampe D. Zeisberg M. (2014). Potential approaches to reverse or repair renal fibrosis. Nat. Rev. Nephrol. 10 (4), 226–237. 10.1038/nrneph.2014.14 24514753

[B68] Tang P. M. Zhang Y. Y. Xiao J. Tang P. C. Chung J. Y. Li J. (2020). Neural transcription factor Pou4f1 promotes renal fibrosis via macrophage-myofibroblast transition. Proc. Natl. Acad. Sci. U. S. A. 117 (34), 20741–20752. 10.1073/pnas.1917663117 32788346 PMC7456094

[B69] Tang P. M. Zhou S. Li C. J. Liao J. Xiao J. Wang Q. M. (2018). The proto-oncogene tyrosine protein kinase Src is essential for macrophage-myofibroblast transition during renal scarring. Kidney Int. 93 (1), 173–187. 10.1016/j.kint.2017.07.026 29042082

[B70] Torres A. Munoz K. Nahuelpan Y. Ap R. S. Mendoza P. Jara C. (2020). Intraglomerular monocyte/macrophage infiltration and macrophage-myofibroblast transition during diabetic nephropathy is regulated by the A(2B) adenosine receptor. Cells 9 (4), 1051. 10.3390/cells9041051 32340145 PMC7226348

[B71] Vierhout M. Ayoub A. Naiel S. Yazdanshenas P. Revill S. D. Reihani A. (2021). Monocyte and macrophage derived myofibroblasts: is it fate? A review of the current evidence. Wound Repair Regen. 29 (4), 548–562. 10.1111/wrr.12946 34107123

[B72] Wang N. Deng Y. Liu A. Shen N. Wang W. Du X. (2017). Novel mechanism of the pericyte-myofibroblast transition in renal interstitial fibrosis: core fucosylation regulation. Sci. Rep. 7 (1), 16914. 10.1038/s41598-017-17193-5 29209018 PMC5717002

[B73] Wang S. Meng X. M. Ng Y. Y. Ma F. Y. Zhou S. Zhang Y. (2016). TGF-β/Smad3 signalling regulates the transition of bone marrow-derived macrophages into myofibroblasts during tissue fibrosis. Oncotarget 7 (8), 8809–8822. 10.18632/oncotarget.6604 26684242 PMC4891006

[B74] Wang Y. Li Y. Chen Z. Yuan Y. Su Q. Ye K. (2022). GSDMD-dependent neutrophil extracellular traps promote macrophage-to-myofibroblast transition and renal fibrosis in obstructive nephropathy. Cell Death Dis. 13 (8), 693. 10.1038/s41419-022-05138-4 35941120 PMC9360039

[B75] Wei J. Xu Z. Yan X. (2022). The role of the macrophage-to-myofibroblast transition in renal fibrosis. Front. Immunol. 13, 934377. 10.3389/fimmu.2022.934377 35990655 PMC9389037

[B76] Wight T. N. Potter-Perigo S. (2011). The extracellular matrix: an active or passive player in fibrosis? Am. J. Physiol. Gastrointest. Liver Physiol. 301 (6), G950–G955. 10.1152/ajpgi.00132.2011 21512158 PMC3233785

[B77] Wu C. F. Chiang W. C. Lai C. F. Chang F. C. Chen Y. T. Chou Y. H. (2013). Transforming growth factor β-1 stimulates profibrotic epithelial signaling to activate pericyte-myofibroblast transition in obstructive kidney fibrosis. Am. J. Pathol. 182 (1), 118–131. 10.1016/j.ajpath.2012.09.009 23142380 PMC3538028

[B78] Yamamura Y. Iwata Y. Furuichi K. Kato T. Yamamoto N. Horikoshi K. (2022). Kif26b contributes to the progression of interstitial fibrosis via migration and myofibroblast differentiation in renal fibroblast. FASEB J. 36 (11), e22606. 10.1096/fj.202200355R 36250931

[B79] Yang J. Wang M. Zhu F. Sun J. Xu H. Chong Lee Shin O. L. (2019). Putative endothelial progenitor cells do not promote vascular repair but attenuate pericyte-myofibroblast transition in UUO-induced renal fibrosis. Stem Cell Res. Ther. 10 (1), 104. 10.1186/s13287-019-1201-5 30898157 PMC6429829

[B80] Yang M. Liu J. W. Zhang Y. T. Wu G. (2021). The role of renal macrophage, AIM, and TGF-β1 expression in renal fibrosis progression in IgAN patients. Front. Immunol. 12, 646650. 10.3389/fimmu.2021.646650 34194427 PMC8236720

[B81] Yang X. Letterio J. J. Lechleider R. J. Chen L. Hayman R. Gu H. (1999). Targeted disruption of SMAD3 results in impaired mucosal immunity and diminished T cell responsiveness to TGF-beta. EMBO J. 18 (5), 1280–1291. 10.1093/emboj/18.5.1280 10064594 PMC1171218

[B82] Yang Y. Duan W. Jin Z. Yi W. Yan J. Zhang S. (2013). JAK2/STAT3 activation by melatonin attenuates the mitochondrial oxidative damage induced by myocardial ischemia/reperfusion injury. J. Pineal Res. 55 (3), 275–286. 10.1111/jpi.12070 23796350

[B83] Yi Y. Ma J. Jianrao L. Wang H. Zhao Y. (2018). WISP3 prevents fibroblast-myofibroblast transdifferentiation in NRK-49F cells. Biomed. Pharmacother. 99, 306–312. 10.1016/j.biopha.2018.01.005 29353205

[B84] Zeisberg E. M. Potenta S. E. Sugimoto H. Zeisberg M. Kalluri R. (2008). Fibroblasts in kidney fibrosis emerge via endothelial-to-mesenchymal transition. J. Am. Soc. Nephrol. 19 (12), 2282–2287. 10.1681/ASN.2008050513 18987304 PMC2588112

[B85] Zhao Y. Qiao X. Tan T. K. Zhao H. Zhang Y. Liu L. (2017). Matrix metalloproteinase 9-dependent Notch signaling contributes to kidney fibrosis through peritubular endothelial-mesenchymal transition. Nephrol. Dial. Transpl. 32 (5), 781–791. 10.1093/ndt/gfw308 PMC5427520 27566305

[B86] Zhou R. Liao J. Cai D. Tian Q. Huang E. Lü T. (2021). Nupr1 mediates renal fibrosis via activating fibroblast and promoting epithelial-mesenchymal transition. Faseb J. 35 (3), e21381. 10.1096/fj.202000926RR 33617091

